# A Year's Work in the Guy's Hospital Dispensary

**Published:** 1909-03-27

**Authors:** 


### A YEAR'S WORK IN THE GUY'S HOSPITAL DISPENSARY.

The annual report of Mr. Finnemore, the head of the dispensary staff at Guy's Hospital, to the Governors of that institution on the work of the dispensary and pharmaceutical laboratory for 1908 is full of facts and figures which are highly interesting both to the medical profession and to hospital managers. This report is summarised in the *Guy's Hospital Gazette* for February 20, from which we take the following statistics. It may first be noted that the drug and dressing bills show a total diminution of over £200 compared with those for the year 1907. As the work of the institution is certainly not less than it was, this must mean economy of use or more advantageous buying, or both. The section of the report dealing with anæsthetic drugs is interesting. Chloroform has declined from 881 lbs. to 720 lbs.; whereas ether has jumped from 674 lbs. to 1,008 lbs. We cannot help associating these significant figures with the question of fatalities during 1907 and the appointment of two new anæsthetists as the result of the consideration of this matter by a committee. Ethyl chloride was consumed in twice the quantity previously used; and nitrous oxide gas leaped up over 40 per cent. Probably the increased use of ether accounts for much of these increments. On the other hand cocaine and eucaine declined considerably.

The prevailing fashions in antiseptics are also noteworthy. Of carbolic acid but 20 cwt. was used as opposed to 30 cwt. in 1907. Mercuric chloride declined from 85 lbs. to 56 lbs.; formaldehyde from 672 lbs. to 412 lbs. Boric acid and iodoform were practically stationary. Cresol was used in more than twice the quantity bought in the previous year, and hydrogen peroxide showed also a substantial increase. The favourite of the coal-tar antiseptics, however, is still lysol, of which 680 gall. were used, as against 575 gall. previously. Of drugs for internal consumption bismuth seems to be going somewhat out of use, whereas acetyl salicylic acid is being ordered in rapidly increasing quantities. The report points out that the cost of this drug in 1908 was £14 0s. 3d., whereas if the medical staff had prescribed one particular make of it under the trade name of aspirin the price would have been about £130. Of sera the cost of antidiphtheritic showed a marked decline; the antistreptococcic as marked an increase. As, however, the quantities used are not specified, fluctuations of price may account for some of the variation. The extent to which market quotations affect a hospital drug bill is well shown in the case of glycerine. The amount used, 32 cwt., was only 3 cwt. in excess of the previous year's supply, but the prices were £102 and £76 respectively.

